# Evaluation of the Digisonic® SP cochlear implant: patient outcomes and fixation system with titanium screws

**DOI:** 10.5935/1808-8694.20120034

**Published:** 2015-10-20

**Authors:** Guilherme Machado de Carvalho, Alexandre Caixeta Guimarães, Fabiana Danieli, Lúcia Cristina Beltrame Onuki, Jorge Rizzato Paschoal, Walter Adriano Bianchini, Arthur Menino Castilho

**Affiliations:** a MSc in Medicine, MD, ENT (Fellow in Otology. UNICAMP); b MD. (ENT Medical Resident. UNICAMP); c MSc in Bioengineering. (Specialist in Cochlear Implants); d Speech and Hearing Therapist. (Specialist in Cochlear Implants. UNICAMP); e MD, PhD, ENT. (Professor of Otorhinolaryngology. Head of the Otology Service. UNICAMP); f MSc in Medicine. MD, ENT. (Head and Coordinator of the Otology, Audiology, Cochlear Implant, and Implantable Hearing Aid Service. UNICAMP); g MD, PhD, ENT. (Head and Coordinator of the Otology, Audiology, Cochlear Implant, and Implantable Hearing Aid Service. UNICAMP); Otology, Audiology and Implantable Ear Prostheses Ear, Nose, Throat and Head & Neck Surgery Department

**Keywords:** cochlear implantation, deafness, electrodes, implanted, hearing loss sensorineural, prostheses and implants

## Abstract

Cochlear implants have revolutionized the way patients affected by severe hearing loss experience the world. Neurelec developed a fixation system with two titanium screws that requires no skull bone drilling.

**Objective:**

To describe the outcomes and procedure-related details of a series of patients implanted with the Digisonic® SP cochlear implant.

**Method:**

This retrospective study analyzed patients submitted to cochlear implant placement within a period of 18 months. All patients had postlingual hearing impairment. Data was collected from patient charts and standard questionnaires answered by the surgeons in charge of carrying out the procedures.

**Results:**

The six patients offered the Digisonic® SP cochlear implants were operated by experienced surgeons. The procedures took 95 to 203 minutes (mean = 135') to be completed, which is less time than what has been described for other fixation approaches. No complications were recorded and hearing improvement was satisfactory.

**Conclusion:**

The Digisonic® SP cochlear implant developed by Neurelec offered good audiological results for adult patients, shorter surgery time, and no surgical or postoperative complications.

## INTRODUCTION

Cochlear implants have revolutionized the way patients affected by severe hearing loss experience the world. Good outcomes with improved speech perception in patients of all age ranges have been observed^1^, ^2^, ^3^. Cochlear implants improve patient quality-of-life even more significantly in subjects aged 65 years and older^4^.

The implantation procedure is safe and reliable, but complications occur in about 16% of the patients. The most frequent adverse event relates to the insertion of electrodes into the cochlea, seen in about 4% of the cases^5^.

Migration of internal components has been described by many authors and may lead to cochlear implant malfunction and local infection^6^, ^7^, ^8^. Various implantation procedures have been described in the literature, with less invasive approaches gaining significant attention^9^, ^10^, ^11^.

The production of the bed on which the internal component is positioned is an important and onerous stage in the procedure, and has been the target of significant investment by cochlear implant makers^12^, ^13^. More is thus spent in the development of surgical instruments and new materials such as titanium plates, propylene mesh, GORE-TEX, absorbable materials, and others^14^, ^15^, ^16^.

**Figure 1:**
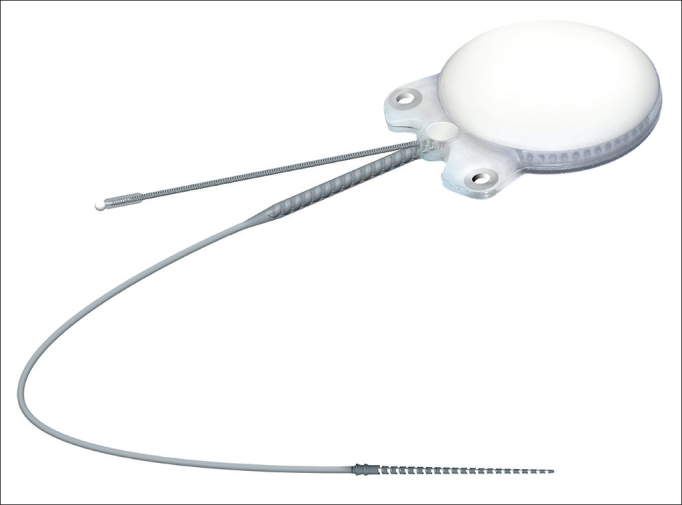
Internal component with receptor, stimulator, and magnet, in a small sealed ceramic framework on a titanium base plate. Lateral niche has two fixation screws - Digisonic® SP developed by Neurelec.

In regards to internal component fixation, Neurelec Inc. (Sophia-Antipolis, France) developed a fixation system with two titanium screws that requires no skull bone drilling^17^ (Figure 1).

Therefore, this study aimed to describe the outcomes and procedure-related details of a series of patients implanted with the Digisonic® SP cochlear implant and discuss the postoperative clinical and audiological findings observed in the last 18 months.

## METHOD

This retrospective study was carried out in a specialized tertiary care hospital. Patients implanted with Digisonic® SP cochlear devices were assessed for a period of 18 months.

A digital data collection protocol was designed. The following parameters were analyzed: age, gender, hearing loss etiology, time with hypacusis, side of implantation, pre and postoperative audiometric data (audiometry and speech perception testing), length of surgery, time to fixate the internal component, surgery complications, follow-up time in months. All patients in this series had post-lingual hearing loss (hearing was lost after they had developed speech and language skills).

Data was collected from patient charts, and a standard questionnaire answered by the surgeons who carried out the procedures.

The identity of the patients was not disclosed, as required by the ethical principles of the institution in which the study was conducted.

### The patients

The patients selected for the study had Digisonic® SP cochlear devices implanted within a period of 18 months. All subjects followed a preoperative protocol in which the etiology of the impairment was investigated through lab workup, genetic tests, CT and MRI scans of the ears and mastoids, psychological evaluation, and thorough speech and hearing assessment.

### The device

The Digisonic® SP cochlear implant system - made up by cochlear implant Digisonic® SP and speech processors Digi SP or Digi SP'K - was launched by French company Neurelec S.A. in 2004. This device belongs to the latest generation of implantable components developed by Neurelec and offers several advancements in relation to previous generation devices. The device's main features include an increased number of electrodes in the beam to allow for a greater number of active channels for stimulation and better spectral representation inside the cochlea, and a fixation system for the receiver-stimulator that uses two titanium screws and raises the stimulation rate through the “*Mean Peak Interleaved Sampling*” (MPIS) sound processing strategy.

#### Device internal component

Internal component Digisonic® SP developed by Neurelec is shown in Figure 1.

The receiver-stimulator (RS) is made up of a convex ceramic capsule, a titanium base plate, both coated with biocompatible silicone. Digisonic® SP is extremely compact, and both its electronic components used in signal decoding and its internal magnet are comprehended by one single structure with a diameter of 30 mm called the monoblock.

Table 1 describes the characteristics of Digisonic® SP.

**Table 1:** Digisonic® SP technical features.

Features	Observations
Indication	Normal or ossifed cochlea
Mechanical properties	
Receptor size	4.9 mm border – 5.75 mm center – 30.2 mm diameter
Weight	10.5 g
Receptor materials	Titanium base plate – A1203 ceramic capsule – silicone envelope
Stimulation	
Mode of stimulation	Monopolar or common ground - two-phase pulses
Frequency of stimulation	Up to 2400 pulses per second
Pulse duration	1 to 120 μs (resolution = 0.5 μs)
Pulse amplitude	Adjustable
Electrode impedance	Under 2kΩ
Electrode coupling capacity	Mean residual current under 100 nA
Safety	
Surgery	Fixation with two titanium screws with bone adhesion
Electrode depth of insertion	25 mm
Cochleostomy	1 mm
MRI compatibility	Compatible (1.5 Tesla)
Reference electrodes (ground)	2
Electrode beam	
Materials	Platinum-iridium, silicone
Active electrodes	20
Electrode beam active length	25 mm
Base diameter (silicone ring)	1.07 mm
Apex diameter (distal end)	0.5 mm
Electrode surface	0.63 mm2 to 1.1 mm2
Type of electrode beam	Straight with memory
Objective measurements	
Allows measurement of electrically evoked brainstem response (EABR), stapedial refexes, and psychoacoustic tests (such as the gap test etc). USB Digistim® SP diagnostic system (for Windows 98SE, ME, 2000, XP and VISTA®)	

**Figure 2:**
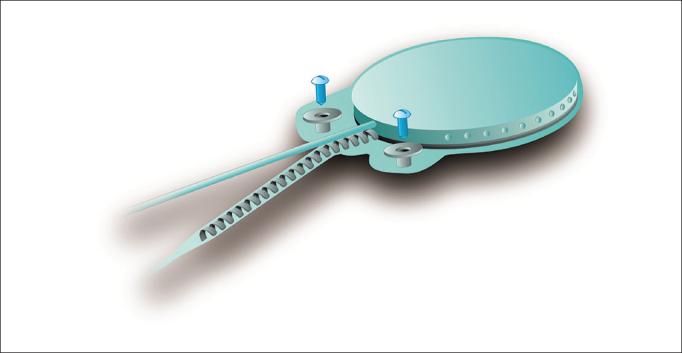
Digisonic® SP fixation system.

**Figure 3:**
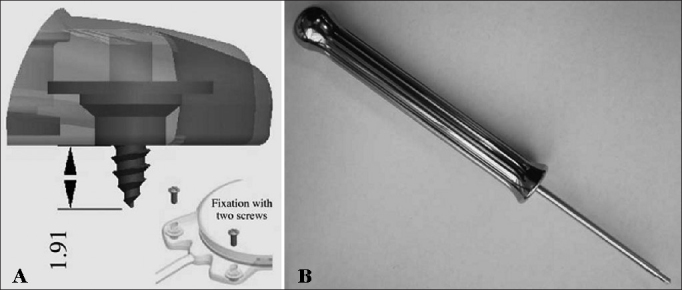
Digisonic® SP fixation system with two titanium screws driven 1.91 mm into the temporal bone. There is no need to drill or prepare the site. Developed by Neurelec S.A.

The device's fixation is done by two 3.4 mm titanium screws bolted into two small silicone-coated titanium orifices with 5 mm in diameter positioned in the ends of the receiver-stimulator, as shown in Figures 2 and 3. The screws are driven 1.91 mm into bone tissue^17^.

The compact structure and the fixation screws of the Digisonic® SP allow for quicker, less invasive surgery without the need to drill or suture bone tissue to position or fixate the implant^17^.

Digisonic® SP has atraumatic flexible screws that adjust quickly to the site in which they were positioned and connect firmly to the RS. The beam is made of 20 platinum-iridium electrodes, which allow the stimulation of up to 20 channels along the cochlea, with an active length of 25 mm and a stimulation rate of up to 1,000 pulses per second for each stimulation channel enabled by processing strategy MPIS. It is also equipped with silicone rings to facilitate insertion^18^.

The internal device contains a two-way telemetry system to record electrode impedance. Electrode impedance is recorded through diagnostic interface Digistim SP and software program Digistim for Windows SP® version 1.9.15, in which it is also possible to perform other objective measurements such as electrically evoked auditory brainstem responses (EABR) and electrically evoked stapedius reflex thresholds (ESRT)^18^.

EABR measurement is carried out routinely during surgery to verify device function and the stimulation of peripheral auditory nerve fibers^19^. In addition to that, EABR can also be used to predict the psychophysical levels to program the speech processor^20^, a particularly important step in the treatment of pediatric patients. EABR is measured at the end of surgery with the patient still under general anesthesia.

**Figure 4:**
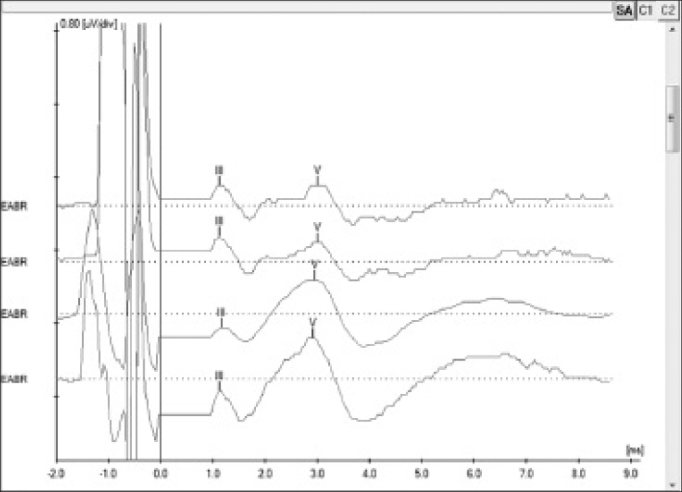
Electrically evoked brainstem responses measured during surgery for subject 5 electrodes 19, 14, 9 and 6 using Interface Navigator Pro and Software AEP version 7.0.0, Biologic. Waves III and V can be seen, showing proper nerve fiber stimulation.

The procedure comprehends the electrical stimulation of nerve fibers through the electrodes inserted in the cochlea and the acquisition of responses through a conventional BAEP measurement device and a synchronization cord. It is possible to view waves II, III, and V, the last two being the most commonly observed; wave V is indicative of effective cochlear nerve stimulation. The absolute latencies of these waves are reduced when compared to conventional BAEP, given that the stimulation on EABR is performed directly in the cochlear nerve through the electrodes in the implant beam^20^ (Figure 4).

Patients using the Digisonic® SP implant can undergo magnetic resonance imaging in scanners of up to 1.5 Tesla, if recommendations are followed^18^.

## RESULTS

Table 1 shows the characteristics and technical details of the device used in the described procedures.

Tables 2 and 3 show general and specific data of the patients implanted with Digisonic® SP.

**Table 2:** General implant patient data.

Subject	Gender	Age*	Time with dysacusis*	Dysacusis etiology	Date of CI	Date of activation	Side of CI
1	M	50	25**	Infection	12/04/11	31/05/11	Left
2	F	33	21	Idiopathic (Progressive)	23/10/10	01/12/10	Right
3	F	30	10	Ototoxicidade	28/09/11	25/10/11	Left
4	F	52	15	Idiopathic	30/08/11	25/10/11	Left
5	M	20	9	Idiopathic (Progressive)	20/03/12	26/14/12	Right
6	F	26	20	Rubeola	14/12/11	02/02/12	Left

^*^ years;

^**^ right ear dysacusis at one year of age; hearing on left ear worsened at age 25 after meningitis; stroke at age 49 obliterated remaining left ear hearing; M: male; F: female; CI: cochlear implantation; dates on DD/MM/YY format.

**Table 3:** Specific implant patient data.

Subject	Electrode insertion site Cochleostomy	Electrode type	Active channels	External processor Digi SP®	Follow Up*
1	Cochleostomy	Digisonic® SP	All	Digi SP®	15
2	Cochleostomy	Digisonic® SP	All	Digi SP®	33
3	Cochleostomy	Digisonic® SP	All	Digi SP®	10
4	Cochleostomy	Digisonic® SP	All	Digi SP®	11
5	Cochleostomy	Digisonic® SP	All	Digi SP®	4
6	Cochleostomy	Digisonic® SP	All	Digi SP®	8

^*^ months.

Table 4 shows the data on surgery-related occurrences and length of the procedure.

**Table 4:** Surgery implant patient data.

Subject	Total length*	Fixation time*	Time saved*, **	Complications (intra and postoperative)***
1	158	3.58	30	None
2	203	5.60	30	None
3	144	4.12	30	None
4	100	4.68	30	None
5	109	3.44	30	None
6	95	6.01	30	None

^*^ minutes;

^**^ time to fixate internal component as reported by surgeon. The mean time to the completion of this portion of the procedure (production of internal component niche) was calculated by the analysis of 10 random cases done within the same time period using different fixation modes (temporal bone drilling to make the niche);

^***^ fixation errors, bleeding, injured noble structures, infection, dehiscence, internal component migration, electrode migration, need to remove the implant, cholesteatoma, otitis media.

MRI and CT scans did not reveal radiological alterations.

EABR and ERST measurements were performed in all procedures at the end of surgery with the patients still under general anesthesia. Measurement outcomes were satisfactory and showed the device was functioning properly.

**Figure 5:**
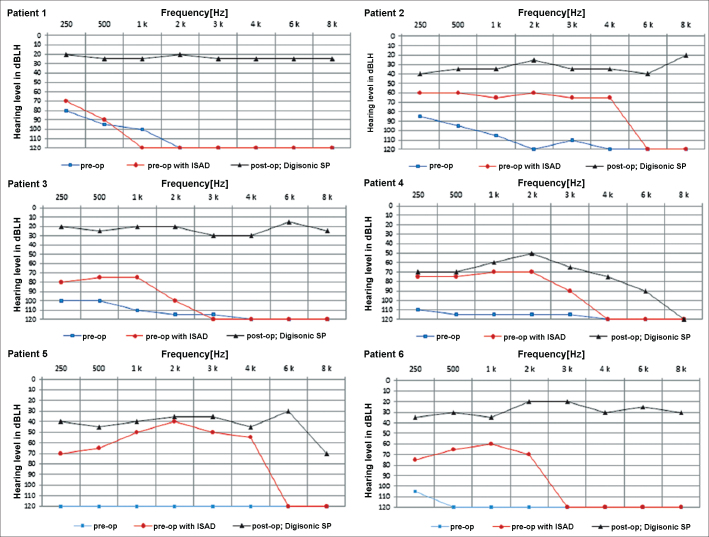
Pre and postoperative audiometric thresholds of six cochlear device implantation patients using Digisonic® SP. Note threshold improvement.

The graphs below show the pre and postoperative audiometric test results (Figure 5).

## DISCUSSION

The Digisonic® SP cochlear implant developed by Neurelec has a fixation system with two titanium screws, which does not require the production of a niche to place the cochlear implant internal component or any drilling on the patient's skull bone. In addition to reducing the risks and complications associated with the production of the cochlear implant niche, this fixation system reduces the length of surgery.

In our study, six patients were implanted with Digisonic® SP by experienced surgeons, and surgery length ranged from 95 and 203 minutes, with a mean length of 135 minutes. The mean length of a conventional cochlear device implantation procedure is 255 minutes in the hands of experienced surgeons using the S-shaped retroauricular incision to make the implant niche, and 200 minutes when using a small retroauricular incision and a subperiosteal pouch^21^. Therefore, length of surgery in our series was shorter than that of procedures using other modes of fixation.

Studies looking into the cost of cochlear implants use a wide array of methods, and cost estimates may vary significantly depending on what is considered as part of the cost^22^. However, according to the literature, the cost of a unilateral implant in a patient with post-lingual hearing loss ranges between € 30,026 (USD 21,018) and € 45,770 (USD 32,039), with the device accounting for most of the cost^23^. In Brazil, the cost of a Digisonic® SP is similar to the cost of a conventional fixation device.

Despite the high cost, the benefits of cochlear implants far outweigh the costs as they enable hearing rehabilitation, improved communication, and better quality-of-life for deaf patients, with even more evident results seen in younger patients^23^, ^24^, ^25^.

The patients in our series had no complications, and the level of auditory gain was satisfactory in all cases. In general terms, mean complication rates of 16% have been described in cochlear device implantation procedures^5^. Despite the small size of our sample, our results were better than average.

## CONCLUSION

The Digisonic® SP cochlear implant developed by Neurelec presented good audiological outcomes in our series, shorter length of surgery, and no intra or postoperative complications.
